# Features of the Solid HDDR Process in Sintered (Nd,Pr,Gd)-Fe-B Magnets at Low Hydrogen Pressure and Low Temperature

**DOI:** 10.3390/ma18174019

**Published:** 2025-08-27

**Authors:** Renhui Liu, Ihor I. Bulyk, Munan Yang, Yifan Wang, Hang Wang

**Affiliations:** 1 Jiangxi Province Key Laboratory of Magnetic Metallic Materials and Devices, Jiangxi University of Science and Technology, Ganzhou 341000, China; liurenhui@jxust.edu.cn (R.L.); yangkl930@163.com (M.Y.); wanghang@jxust.edu.cn (H.W.); 2 National Rare Earth Functional Materials Innovation Center, Ganzhou 341000, China; 3 Key Laboratory of Development and Application of Ionic Rare Earth Resources, Ministry of Education, Ganzhou 341000, China

**Keywords:** solid disproportionation, recombination, sintered magnets, phase composition, texture

## Abstract

This article investigates the connection between the process parameters of solid hydrogenation, disproportionation (HD), desorption, and recombination (DR) (HDDR) in sintered (Nd,Pr,Gd)-Fe-B magnets, as well as their phase composition and degree of texture (DoT). During HD, hydrogen pressures of 10–50 kPa were applied at temperatures ranging from 700 to 785 °C for reaction times ranging from 3 to 11 h. DR was performed at 750–850 °C. The HD reaction was observed across the full range of hydrogen pressure and temperature. The phase composition of the disproportionation products depends on the depth in the sample. Applying HDDR treatment at a pressure of 10 kPa is an effective way to increase the DoT of magnets. Magnets are anisotropic following the HDDR treatment across the parameter ranges. The dependence of the DoT value on HDDR treatment parameters is complicated, with the main trend being a decline in DoT with increasing hydrogen pressure. The DoT is determined by the disproportionation and recombination temperatures, as well as the depth at 50 kPa pressure. The recombined phase is isotropic near the sample surface and highly anisotropic within the sample after 50 kPa is applied.

## 1. Introduction

Discovered more than forty years ago [1], Nd-Fe-B-based permanent magnets have been widely used in large quantities in computers, consumer electronics, wind turbines, and vehicles [2]. The production volume of these magnets is rapidly increasing and will continue with the rapidly growing demand for environmentally friendly devices such as wind turbines and electric cars [3]. The wide range of applications for permanent magnets is stimulating the search for new magnetic materials [4,5] and for studies aiming to improve the magnetic properties of known materials [6], particularly by grinding their microstructure down to the nanoscale [7,8,9].

Hydrogen treatment, via hydrogenation, disproportionation (HD), desorption, and recombination (DR) (HDDR), has shown promise in grinding the microstructure of ferromagnetic materials to a fine level [10]. The HDDR process is well-known [10,11] and is used to produce anisotropic materials [12]. New applications and features of this method have been found. It can be used to recycle magnets from end-of-life products [13], grind microstructures of ferromagnetic materials to the nanoscale [14,15], and prepare nanocomposites [16,17,18]. HDDR has been applied as a low-temperature method for sintering rare earth–transition metal magnetic materials [19,20]. Grinding microstructures of magnetic materials through the HDDR route has a positive effect on their magnetic properties. For example, the coercivity of SmCo_5_-based powders increases up to *μ*_oJ_*H*_C_ = 4.7 T [21], and the post-sintering treatment of SmCo_5_ magnets increases their coercivity up to ≈5 T [22]. These results make this method highly attractive for post-sintering Nd-Fe-B magnets. The coercivity of Nd-Fe-B-type magnets depends on their grain size, and it increases as the grain size decreases [23]. The conventional powder metallurgy approach of producing sintered magnets from fine powder and, consequently, with a fine microstructure, is exhausted because of the powder oxidation. The HDDR treatment is the clear path forward for creating fine microstructures in sintered Nd-Fe-B magnets. This method allows for the formation of submicron-scale microstructure in bulk materials through the so-called solid HDDR process [24].

This study aims to investigate the peculiarities of the solid HDDR process at low hydrogen pressure and low temperature and its influence on the phase composition and degree of texture (DoT) of sintered (Nd,Pr,Gd)-Fe-B magnets.

## 2. Materials and Methods

**Figure 1:**
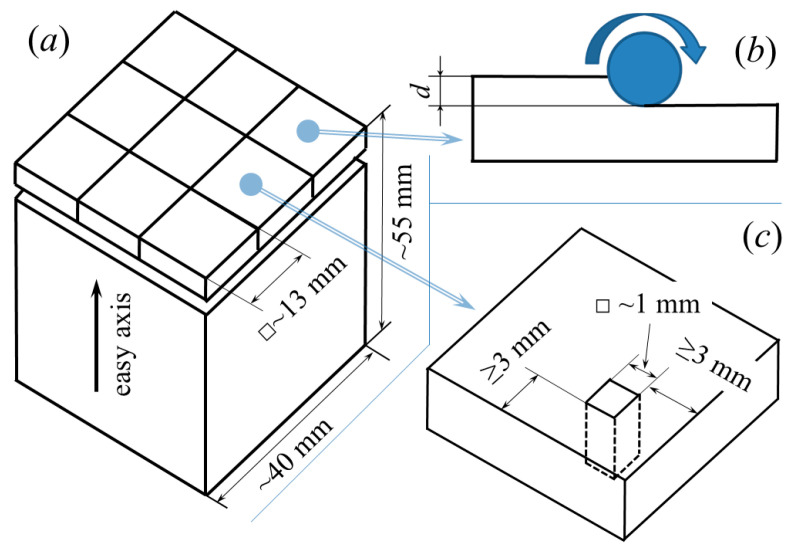
Sample preparation schema for (**a**) HDDR treatment, (**b**) polishing samples to depth *d* for X-ray measurements at this depth, and (**c**) magnetic measurements. ☐~13 mm—a square with a side length of ~13 mm.

**Figure 2:**
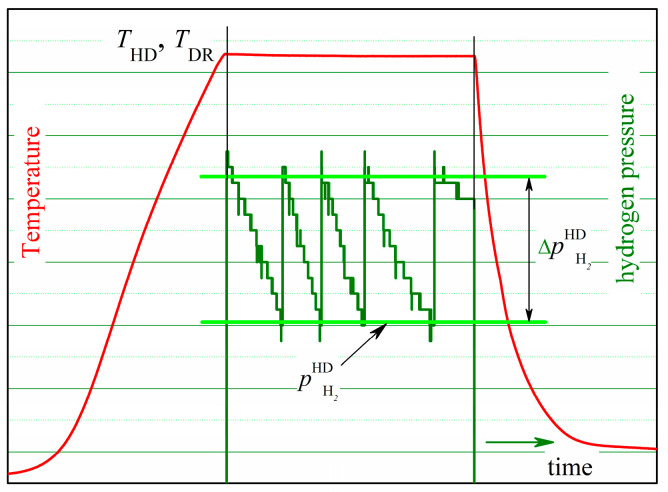
Schematic of HDDR process conditions; T_HD_ and T_DR_ are the temperatures of the disproportionation and recombination reactions, respectively. The desired hydrogen pressure during HD is $p_{H2}^{HD}$. The excess pressure value ${\Delta p}_{H2}^{HD}\approx$ 1.0–1.2 kPa compensates for the drop in hydrogen pressure caused by hydrogen absorption.

This study used commercial R-Fe-B-type sintered magnets supplied by Ganzhou Fortune Electronics Co. Ltd., Ganzhou, China, wherein R—(Nd,Pr,Gd), with the following nominal composition (in wt%): R—31.2; B—0.88; Cu—0.1; Al—0.7; Co—1; Ga—0.15; Zr—0.15; Fe—65.82. The starting magnets were parallelepiped-shaped with a base of ~40 mm and a height of ~55 mm. The magnetic easy axis was along the longer side of the magnet. The solid HDDR technique [24] was used to treat plate-shaped magnet samples measuring ~13 × ~13 × ~2 mm^3^ that were cut using the electro-spark erosion method, as shown in Figure 1a. The rig for the HDDR treatment is described elsewhere [25]. Disproportionation and recombination were performed in two separate steps, with heating, keeping, and cooling according to the schema in Figure 2. During the HD treatment, the material batch included four samples with a total material weight of ~10.0–10.5 g. The HD reaction was performed at hydrogen pressures ($p_{H2}^{HD}$) of 10, 20, 30, and 50 kPa. The change in $p_{H2}^{HD}$ during HD is shown in Figure 2. The duration (τ) of the HD reaction was *τ* = 3.5 and 6 h when $p_{H2}^{HD}$ = 10 kPa, *τ* = 3 h when $p_{H2}^{HD}$ = 30 kPa, and *τ* =2.5, 4.5, and 6 h when $p_{H2}^{HD}=$ 50 kPa. In addition, when $p_{H2}^{HD}$ = 20, 30, and 50 kPa, the HD treatment was carried out for different durations. Specifically, the hydrogen pressure should not decrease for at least 30 to 45 min prior to considering the disproportionation reaction complete. The temperatures of the disproportionation reaction were 700, 715, 750, and 785 °C, chosen to be within the disproportionation zone of the pressure–composition–temperature relationship in the R-Fe-B alloy–hydrogen gas system [25].

**Figure 3:**
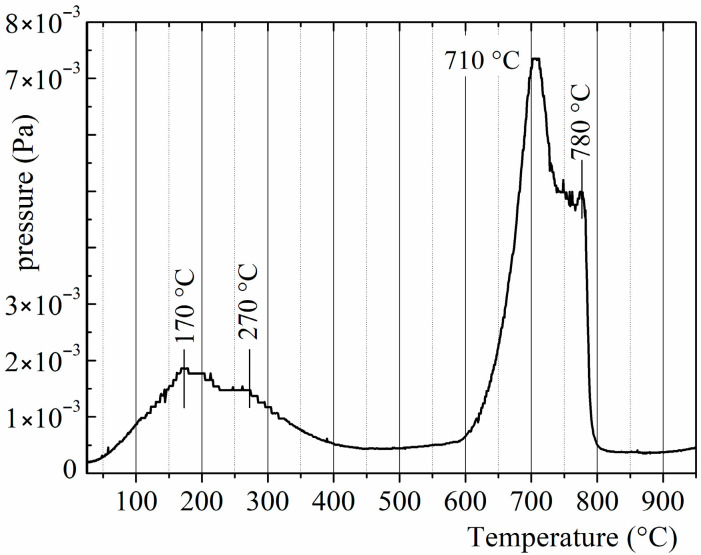
Hydrogen desorption curve during DR in R-Fe-B powders.

The batch of magnets for DR consisted of no more than eight samples, including one of four samples from several batches treated using the HD route. The temperatures of the DR reaction, *T*_DR_ = 750, 810, and 850 °C, were chosen according to the data for the temperature of hydrogen desorption from disproportionated R-Fe-B powder (Figure 3). The DR reaction time depends on the temperature. Samples were heated up to *T*_DR_ and kept at that temperature until the pressure in the chamber reached (2–4) × 10^−4^ Pa.

The phase composition was analyzed using X-ray diffraction (XRD) data collected with Bruker (D8 Advance, Bruker, German, Karlsruhe) and PANalytical instruments with Cu-K_α_ radiation sources (Malvern Panalytical, Malvern, UK). The X-ray patterns for the plates were measured for the sample surface, after grinding the oxide layer, and at different depths. The samples were ground to the required depths using grinding paper before measuring XRD patterns at different depths (Figure 1b). The counting time during the measurements was 60–240 min. The PowderCell 2.2 software package was used for phase identification [26]. The magnetic properties of parallelepiped-shaped samples (~1 × ~1 × ~2 mm^3^, Figure 1c) were measured using the Physical Property Measurement System (DynaCool, PPMS). The anisotropy of the HDDR-treated magnets was determined by the degree of texture, defined as (*M*_r_ (||) − *M*_r_ (⊥))/*M*_r_ (||) × 100%, where *M*_r_ (||) and *M*_r_ (⊥) [27] denote the remanence along the directions parallel and perpendicular to the alignment direction, respectively.

## 3. Results

### 3.1. Phase Composition of Starting Magnets

**Figure 4:**
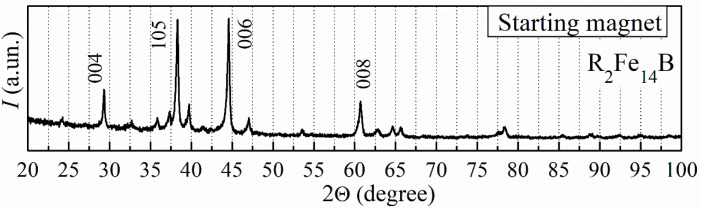
The XRD pattern of the sintered R-Fe-B magnets (measured for the sample surface).

Only the R_2_Fe_14_B-type ferromagnetic phase was revealed by the XRD measurement for the surface of the starting sintered magnets (Figure 4). The increased intensities of crystallographic peaks (004), (105), (006), and (008) for this phase indicate that the starting magnets were textured with the crystallographic axis c oriented in one direction.

Hereinafter, the Miller indices of the R_2_Fe_14_B phase peaks with the highest intensity in the textured material will be marked in the figures. Other R_2_Fe_14_B phase indices will remain unmarked.

### 3.2. Interaction of Sintered Magnets with Hydrogen at 10 kPa

**Figure 5:**
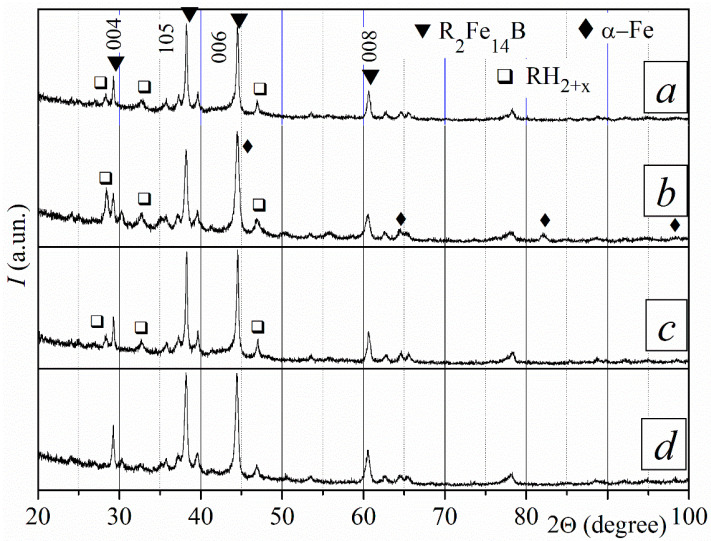
XRD patterns of sintered R-Fe-B magnets at various depths (*d*) after different stages of the HDDR process. Conditions of the HD stage: hydrogen pressure of 10 kPa; temperature of 715 °C. After HD: (**a**) *τ* = 3.5 h, *d* = 410 µm; *τ* = 6 h: (**b**) *d* = 0 µm and (**c**) *d* = 400 µm; (**d**) after HDDR, *d* = 0 µm.

At the HD stage, the sintered magnets were treated in hydrogen at a pressure of 10 kPa and a temperature of 715 °C. This is the temperature at which the disproportionation reaction rate is the highest [25]. The magnets were held at this temperature for 3.5 and 6 h. The phase composition of the magnets after treatment depends on the treatment duration. After interacting with hydrogen for 3.5 h, the phase composition does not depend on the depth in the sample. XRD measurements show the presence of a small amount of a rare earth hydride (RH_2–3_) in addition to the initial ferromagnetic phase R_2_Fe_14_B (Figure 5a). The presence of rare earth hydride RH_2–3_ in the XRD pattern indicates that the rare earth-rich phase disproportionates only under these conditions. The RH_2–3_ peaks were only revealed because the R-rich phase contains significantly more R elements than Fe.

The phase composition of the magnet after its interaction with hydrogen for 6 h depends on the depth in the sample. A small amount of RH_2–3_ and α-Fe was revealed near the surface of the sample (Figure 5b). This indicates that the disproportionation of the main ferromagnetic phase occurs after that of the rare-earth-rich phase. The phase composition inside the sample includes the rare earth hydride and the R_2_Fe_14_B phase (Figure 5c).

Heating magnets in a vacuum during DR, following their treatment during the HD stage for *τ* = 3.5 and 6 h, restores the initial phase composition for both cases (Figure 5d).

### 3.3. Interaction of Sintered Magnets with Hydrogen at 20 kPa

**Figure 6:**
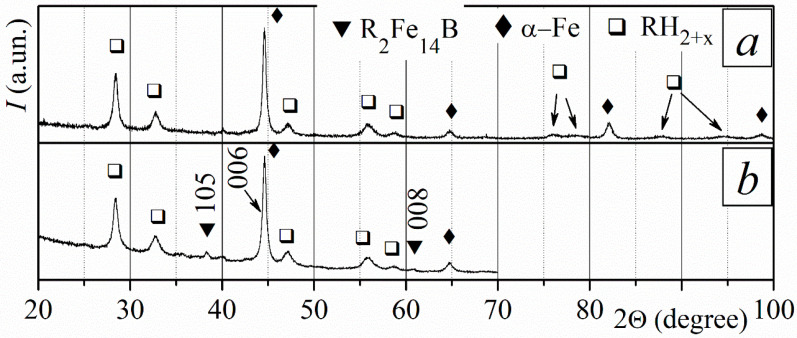
XRD patterns of sintered R-Fe-B magnets at various depths (*d*) after different stages of the HDDR process. Conditions of the HD stage: hydrogen pressure, 20 kPa; temperature, 750 °C; and reaction time, until the alloy completes hydrogen absorption. After HD: (**a**) *d* = 350 µm; (**b**) *d* = 600 µm (the pattern was measured within an angle range of 20–70°).

At a hydrogen pressure of 20 kPa, the HD stage in the sintered magnet was carried out at a temperature of 750 °C, and the magnet was held at this temperature until the sample completed hydrogen absorption. A small difference was revealed amid disproportionation products, depending on the depth of the sample. The main ferromagnetic phase completely disproportionates near the sample surface. The rare earth hydride and iron were detected from the surface of the sample to a depth of up to 350 µm (Figure 6a). Together with two phases of RH_2–3_ and α-Fe, the residuals of the R_2_Fe_14_B ferromagnetic phase are present at a depth of 600 µm (Figure 6b). The small peaks on the experimental XRD pattern in Figure 6b were identified as the (105), (006), and (008) peaks of the R_2_Fe_14_B-type structure because their intensities are increased in textured material with the R_2_Fe_14_B-type phase. Note that the textured initial magnets (Figure 4) were investigated here. After their interaction with hydrogen at different degrees of disproportionation reaction, the remnants of the starting grains of the R_2_Fe_14_B phase preserved their crystallographic orientation from the starting state. The reasons for this form the basis for the conclusion that the very low-intensity peaks in the XRD pattern, which coincide with the (105), (006), and (008) peaks of the R_2_Fe_14_B phase, also belong to that phase. Similar diffractograms presented in this paper are interpreted in the same way.

The DR stage results in the complete reformation of the R_2_Fe_14_B ferromagnetic phase at all depths in the sample. According to the XRD patterns, the magnet is textured after the recombination at all temperatures.

### 3.4. Interaction of Sintered Magnets with Hydrogen at 30 kPa

With the aim of producing textured R-Fe-B-type materials through the HDDR route, the hydrogen pressure of 30 kPa is very often applied at the first stage of the treatment. In this work, the solid HDDR process was studied in sintered magnets at a pressure of 30 kPa at temperatures of 715 and 755 °C. These temperatures are lower than those used in the literature data. The two experiments were performed for 3 h until the sample completed hydrogen absorption.

**Figure 7:**
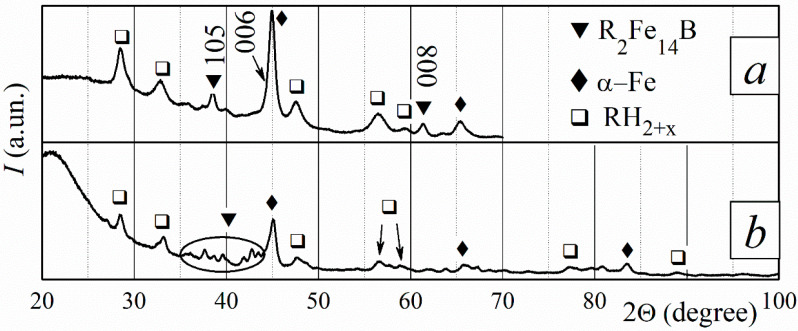
XRD patterns of sintered R-Fe-B magnets after the HD stage, measured for powder and plate samples at various depths (*d*). Conditions of the HD stage: hydrogen pressure of 30 kPa, temperature of 715 °C, and *τ* = 3 h. (**a**) Pattern for a plate at a depth of *d* = 100 µm; (**b**) pattern for random powder. The pattern in (**a**) was measured within an angle range of 20–70°.

The disproportionation reaction is not complete after three hours of interaction between the magnets and hydrogen at a temperature of 715 °C. The XRD patterns obtained for the sample surface (Figure 7a) and for the powder (a part of the bulk sample was ground into a powder (Figure 7b) revealed the disproportionation products and the residues of the main ferromagnetic phase after the HD stage. The XRD pattern measured for the sample surface shows the residuals of the R_2_Fe_14_B phase due to the presence of (004), (105), (006), and (008) peaks with increased intensity. The most intensive peaks of this phase were also revealed in the pattern measured for random powder. The main ferromagnetic phase reforms through the desorption of hydrogen at three DR temperatures. The recombined phase is textured throughout the entire sample.

**Figure 8:**
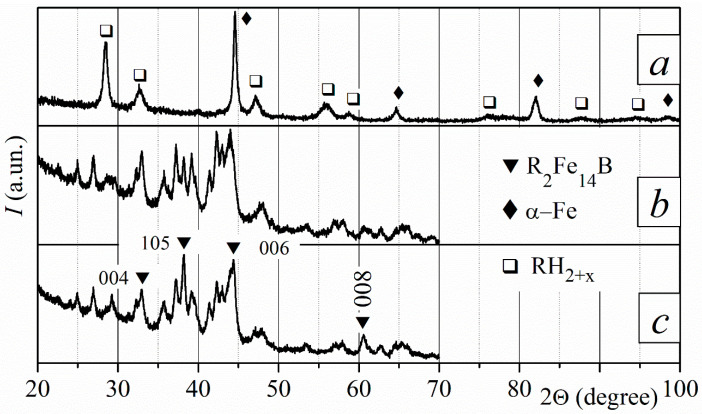
XRD patterns of sintered R-Fe-B magnets at various depths (*d*) after different stages of the HDDR process. Conditions of the HD stage: hydrogen pressure, 30 kPa; temperature, 755 °C; and reaction duration, until the alloy stops absorbing hydrogen. (**a**) After HD, *d* = 50 µm; after HDDR: (**b**) *d* = 70 µm and (**c**) *d* = 190 µm. Patterns (**b**,**c**) were measured within an angle range of 20–70°.

Applying a hydrogen pressure of 30 kPa at 755 °C until the sample finishes hydrogen absorption leads to the complete disproportionation of the magnet. Disproportionation products, RH_2–3_ and α-Fe, were detected from the sample surface to a depth of 600 µm (Figure 8a). The disproportionation products recombine in vacuum at all DR temperatures, and the ferromagnetic phase reforms (Figure 8b). The XRD patterns of the recombined phase are characterized by very wide peaks, indicating that this phase has fine grains. The crystallographic orientation of the R_2_Fe_14_B phase grains is dependent on distance from the sample surface. The magnet is isotropic near the sample surface (Figure 8b). The degree of the texture of the recombined R_2_Fe_14_B phase increases toward the sample center, as the XRD patterns shown in Figure 8c clearly demonstrate.

### 3.5. Peculiarities of the HDDR Process in Sintered Magnets at Hydrogen Pressure of 50 kPa—The Dependence of the Phase—Structure State of the Materials at the Different Depths in the Samples on the Disproportionation Temperature and Reaction Time

The study of the interaction of R-Fe-B magnets with hydrogen under 50 kPa pressure received slightly more attention. We study the solid HDDR route for its use as a post-sintering treatment of Nd-Fe-B-type magnets because we expect high magnetic properties similar to those obtained for SmCo_5_-type magnets [22]. Only this type of HDDR processing was chosen, rather than conventional processing, because it provides bulk magnets without cracks. As is known [28], the reaction rate during the solid HDDR process is slower than during the conventional one. The HD stage of the solid HDDR process is very long at low hydrogen pressure. However, there are experimental results showing that higher hydrogen pressure leads to shorter disproportionation reactions [29,30]. Therefore, the knowledge of the possibility of using higher hydrogen pressure during solid HDDR will be very useful.

HDDR-treated magnets must be anisotropic. Despite the prevailing opinion among researchers regarding the texture memory mechanism in HDDR-treated Nd-Fe-B-type materials [31], we did not reject the well-known suggestion [11] that the residues of the ferromagnetic phase present after the disproportionation reaction cause the texture. Moreover, it has been demonstrated that this mechanism is also present in Sm-Co-based system magnets [15]. The time of the disproportionation reaction is the parameter determining the remnants of the initial starting ferromagnetic phase after the disproportionation stage, which is realized at higher hydrogen pressure. This is why the magnets were treated at 50 kPa during the HD stage for different times, namely, 2.25, 4.5, 6, and ~(8–10) hours. The alloy absorbs hydrogen for about 8–10 h at all disproportionation temperatures before the process is complete.

The temperatures during the HD stage, 700, 750, and 785 °C, were chosen according to the nonequilibrium phase diagram of ferromagnetic alloy–hydrogen gas [25].

This section aims to evaluate the possibility of adjusting the solid HDDR parameters in such a way that higher hydrogen pressure and low disproportionation temperature are used, which will reduce the disproportionation duration and yield anisotropic magnets.

The X-ray measurements for sintered magnets after HD and HDDR, with a recombination temperature of 850 °C, are presented below. These were taken at the surface of the sample and at depths of 70 and 500 microns from the surface.

The research results obtained after the disproportionation of the magnets for 2.25 h are not considered here because all samples were cracked or disintegrated.

#### 3.5.1. Disproportionation Temperature of 700 °C

**Figure 9:**
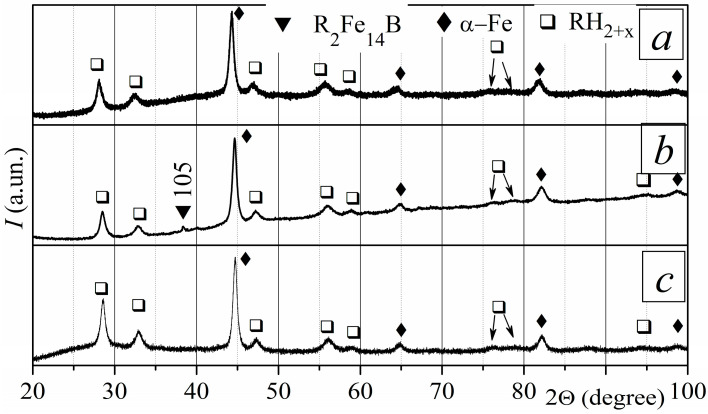
XRD patterns of HD-treated magnets at 700 °C: (**a**) measured at a depth of 70 and (**c**) 500 µm. HD treatment time (*τ*_HD_): (**a**) and (**b**), 4.5 h; (**c**), 8 h.

The disproportionation reaction is complete near the sample surface, at a depth of 70 µm, after the magnets interacted with hydrogen for 4.5, 6.0, and 8.0 h, as shown in Figure 9a. According to the XRD pattern, the crystalline RH_2–3_ and α-Fe phases were revealed in all samples. The broad peaks of these two phases indicate their fine grains. The almost fully disproportionated material was also revealed at a depth of 500 µm. However, there are some differences in the phase composition depending on the reaction time. When the HD dwell time is 4.5 and 6 h, the traces of the R_2_Fe_14_B ferromagnetic phase are revealed in the magnet, along with the disproportionation products (Figure 9b). These traces disappeared after the magnet was maintained at the disproportionation temperature for 8 h (Figure 9c). The grains of disproportionation products are very fine, as evidenced by their broad peaks.

**Figure 10:**
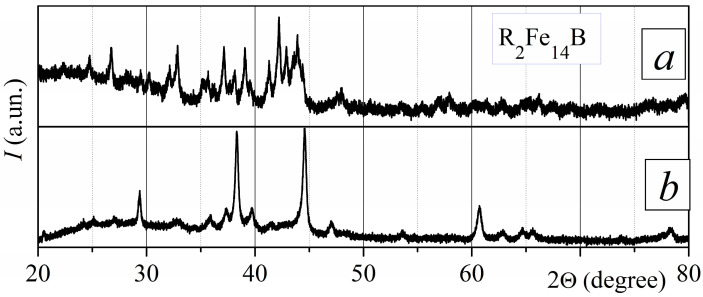
XRD patterns of HDDR-treated magnets at a DR temperature of 850 °C, (**a**) measured at a depth of 70 µm and (**b**) 500 µm. Conditions of the HD stage: hydrogen pressure, 50 kPa; temperature, 700 °C; treatment time (*τ*_HD_), (**a**) 4.5 h and (**b**) 8 h.

The main ferromagnetic phase completely reforms after heating disproportionated samples in vacuum at a temperature of 850 °C (Figure 10a,b). The magnets are isotropic at a depth of 70 µm (Figure 10a) and anisotropic at a depth of 500 µm (Figure 10b).

#### 3.5.2. Disproportionation Temperature of 750 °C

**Figure 11:**
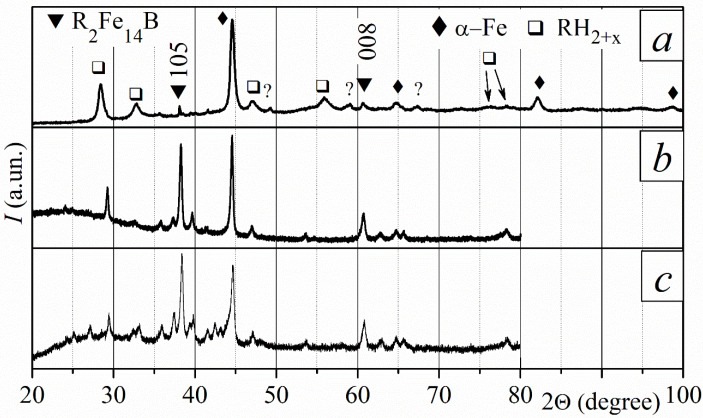
XRD patterns of (**a**) HD-treated magnets at a temperature of 750 °C, and those (**b**,**c**) after HDDR. HD treatment time (*τ*_HD_): (**a**,**b**) 4.5 h; (**c**) 8 h. All patterns were measured at a depth of 500 µm. Patterns (**b**,**c**) were measured within an angle range of 20–80°. “?” marks indicate peaks of an unidentified phase. (Here and in other cases, we associate the presence of an unidentified phase with the partial oxidation of the samples during treatment. We came to this conclusion based on the black color of the sample after conducting some experiments).

After the sintered magnets interacted with hydrogen at a pressure of 50 kPa at 750 °C for 4.5, 6, and 8 h, they disproportionated into rare earth hydride RH_2–3_ and iron α-Fe (Figure 11). The phase composition of the samples is similar for different depths within the range of 70 to 500 µm. The broad peaks of disproportionation products indicate that the materials have fine microstructures. There are differences between samples maintained for 4.5 h at a disproportionation temperature and samples kept for longer times at the same temperature. The magnet treated during the HD stage for 4.5 h contains the remnants of the R_2_Fe_14_B phase. This conclusion is based on the revealed small peaks (105) and (008) of the ferromagnetic R_2_Fe_14_B phase, and their intensities are increased because the remnants of this phase saved the crystallographic orientation of the starting textured sample (Figure 11a). Conversely, the remnants of the ferromagnetic R_2_Fe_14_B phase are absent in the magnet samples treated during the HD stage for 6 and 8 h.

After recombination, the main ferromagnetic R_2_Fe_14_B phase recovered. All magnets contain a main ferromagnetic phase with a slow degree of texture at a depth of 70 µm. The recombined textured R_2_Fe_14_B ferromagnetic phase was revealed at a depth of 500 µm after complete HDDR treatment (Figure 11b,c). The XRD pattern clearly shows that the relative intensity of the (004), (105), (006), and (088) peaks decreased in the sample treated for 8 h compared to the sample treated for 4.5 h during the HD stage. This indicates a reduction in the degree of texture of the magnet, which was treated for 8 h during the HD stage (Figure 11c).

#### 3.5.3. Disproportionation Temperature of 785 °C

**Figure 12:**
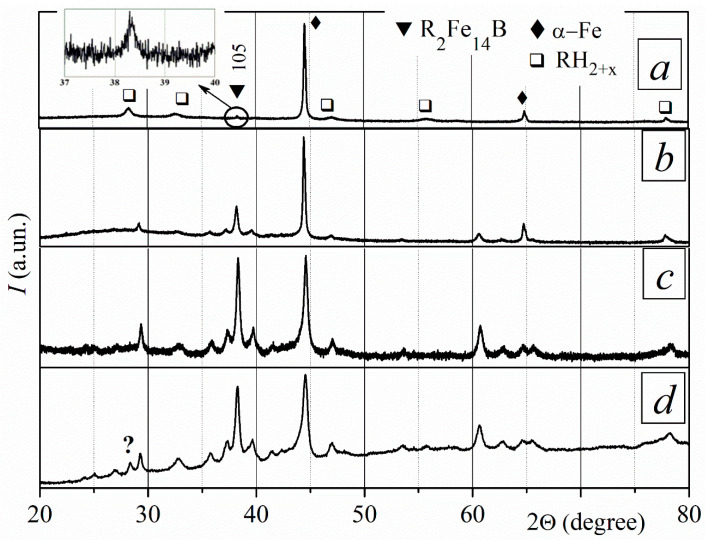
XRD patterns (**a**) of HD-treated magnets at a temperature of 785 °C and (**b**–**d**) after HDDR. HD treatment time (*τ*_HD_): (**a**,**b**) 4.5 h; (**c**) 6 h; (**d**) 8 h. All patterns were measured at a depth of 500 µm. “?” marks indicate peaks of an unidentified phase.

The main ferromagnetic phase of sintered magnets completely disproportionates when interacting with hydrogen at a pressure of 50 kPa and a temperature of 785 °C for 4.5, 6, and 8 h. The products of disproportionation of the R_2_Fe_14_B phase were revealed through XRD measurements at depths of 70 and 500 µm in samples treated with these parameters (Figure 12a). The very small peak at <40° in the XRD pattern of the sample that was disproportionated for 4.5 h coincides with the (105) peak of the textured R_2_Fe_14_B phase. This indicates that remnants of this phase remain in the disproportionated magnet.

All of the magnets that disproportionated after interacting with hydrogen for 4.5, 6, and 8 h recombined when heated in a vacuum at 850 °C. The phase–structure state depends on the duration of the HD reaction and the depth from the surface. At a depth of 70 µm, the sample that was disproportionated for 4.5 h is anisotropic, while the sample that was disproportionated for 8 h is partially anisotropic. At a depth of 500 µm, the main ferromagnetic phase was identified. This phase exhibited varying degrees of texture as a function of disproportionation time. The sample that disproportionated for 4.5 h exhibited the highest DoT (Figure 12b–d).

### 3.6. Magnetic Properties of Sintered R-Fe-B Magnet and Magnet After HDDR

**Figure 13:**
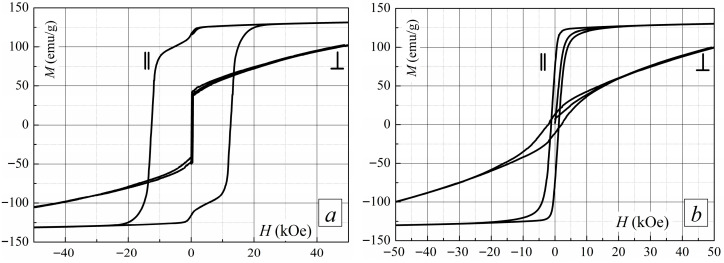
Hysteresis loops for (**a**) as-sintered magnets and (**b**) HDDR-treated magnets. Hysteresis loops measured parallel and perpendicular to axis c are represented by || and ⊥, respectively.

Without optimized heat treatment, the starting sintered magnets exhibit anisotropy and double-phase magnetic behavior (Figure 13a). The magnets remain anisotropic after HDDR treatment (Figure 13b). Their saturation magnetization is similar to that of a sintered magnet. However, their coercivity is drastically reduced because the HDDR parameters, particularly those for the recombination stage, must be optimized. The optimization means that the non-ferromagnetic phase must be formed between main ferromagnetic phase grains, as shown in [32].

**Figure 14:**
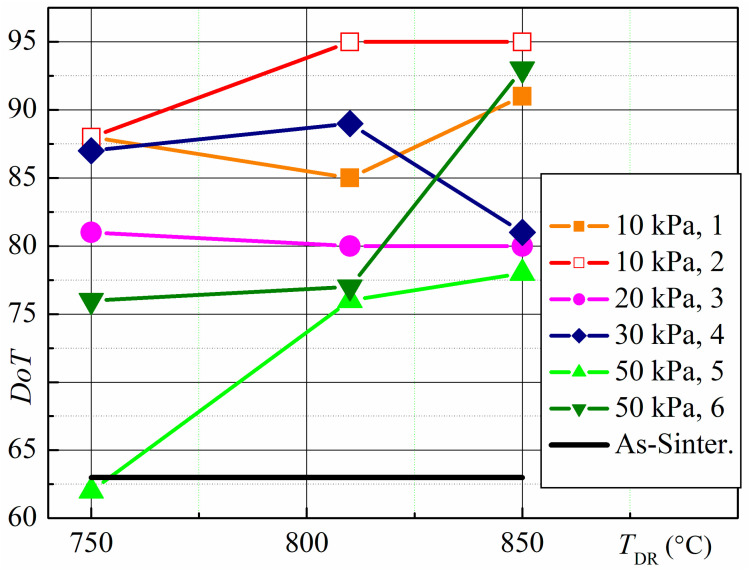
DoT dependence of magnets on HDDR treatment conditions. HD parameters: Hydrogen pressure of 10–50 kPa. Temperature *T*_HD_ and reaction time *ґ*: (1)—715 °C, 3.5 h; (2)—715 °C, 6 h; (3)—750 °C, 11 h; (4)—715 °C, 3 h; (5)—700 °C, 4.5 h; (6)—785 °C, 4.5 h. As-sinter.—for starting sintered magnet; *T*_DR_—the temperature of recombination.

The DoT of the HDDR-treated magnets is significantly greater than that of the as-sintered magnet (Figure 14). The DoT depends on the HDDR parameters. Magnets treated at the lowest hydrogen pressure of 10 kPa have the highest DoT. It was revealed that increasing the disproportionation reaction duration from 3.5 to 6 h and the DR temperature from 750 to 800–850 °C increases the DoT. When the hydrogen pressure is 20 kPa, the DoT is high and nearly independent of the recombination temperature. The DoT is comparable at 30 kPa and 10 kPa; however, it decreases after recombination at 850 °C, when 30 kPa of pressure is applied during the HD stage. At a hydrogen pressure of 50 kPa and a disproportionation time of 4.5 h, the DoT depends on both the disproportionation and recombination temperatures. Using a low disproportionation temperature of 700 °C results in a low DoT. When a pressure of 50 kPa is applied, the DoT is highest when the disproportionation and recombination temperatures are also at their highest (785 and 850 °C, respectively), and it is comparable to that of magnets treated at a hydrogen pressure of 10 kPa.

## 4. Discussion

**Figure 15:**
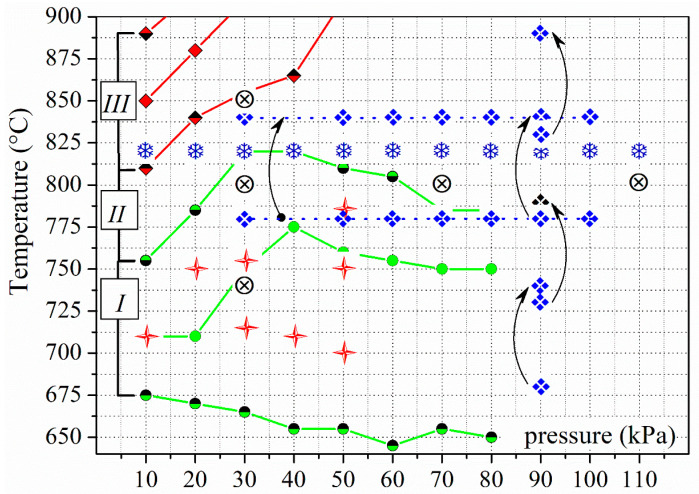
Comparison of the hydrogen pressure—phase composition—temperature schema for the R_2_Fe_14_B-based alloy–hydrogen system [25] (green and red solid lines), as well as the temperatures of the disproportionation reaction and hydrogen pressure according to [28,33,34,35,36,37,38,39,40,41,42,43,44,45,46,47,48,49,50] and this paper. I—The disproportionation reaction zone; II—the interstitial zone; III—the zone of the recombination reaction in hydrogen. ❖—[33,34,35,36,37,38,39]. 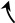
—the transition from low to high temperature during the two-stage treatment. 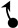
—the transition from 780 to 840 °C at specific pressures during the two-stage treatment. The two-stage treatment involves carrying out the disproportionation reaction for a period of time at temperature *T*_1_ (the first stage) and then for a period of time at temperature *T*_2_ (the second stage). ❄—[40,41,42,43,44]; ⊗—[38,39,40,41,42,43,44,45,46,47,48,49,50]; 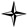
—this work.

A new systematic study was undertaken to investigate the peculiarities of the solid HDDR process in R-Fe-B ferromagnetic materials and to evaluate the feasibility of using this method to treat sintered R-Fe-B magnets. The ultimate goal of this treatment is to improve the magnetic properties of sintered magnets by grinding their microstructure down to the submicron or even nanoscale level. This study evaluates the phase–structure state of the magnets at different stages of treatment, including the phase composition and texture of the main ferromagnetic phase. The main difference in this work from others is that the disproportionation reaction parameters were shifted to the regions with low hydrogen pressure and temperature. This shift was made according to the nonequilibrium ferromagnetic alloy–hydrogen gas phase diagram [25]. Figure 15 clarifies the selection of “the field of working parameters” in this study. The green lines indicate the temperature at the start, peak, and finish of the disproportionation reaction, and the red lines indicate the recombination reaction temperature in hydrogen. The lower and upper green lines are the boundaries of the disproportionation zone. The disproportionation temperatures applied in this research lie within this zone (red stars). The hydrogen pressure and disproportionation reaction temperature values during the HDDR treatment of R-Fe-B-based materials in this study only partially overlap with the values reported in the literature [28,31,33,34,35,36,37,38,39,40,41,42,43,44,45,46,47,48,49,50] (Figure 15).

New data on the peculiarities of the solid HDDR process in sintered R-Fe-B magnets indicate that the disproportionation reaction occurs across the entire 10–50 kPa pressure range when the process is carried out at low hydrogen pressures and temperatures. The degree of the reaction depends on the pressure value and the reaction time.

R-Fe-B materials undergo disproportionation even at a hydrogen pressure as low as 10 kPa. In this case, however, the reaction rate is very slow. After keeping materials for 3.5 h, the rare-earth-rich phase only undoes the disproportionation. Increasing the treatment time to 6 h causes the main ferromagnetic phase to begin disproportionation near the sample surface. These results show that grinding the microstructure of the entire magnet requires too long a treatment time. Nevertheless, applying such low hydrogen pressure increases the degree of texture of the magnets. As can be seen in Figure 14, the DoT of the magnets is highest after their treatment at the lowest hydrogen pressure. We hypothesize that this result is due to the homogeneity of the microstructure of the HDDR-treated magnet near the grain boundary areas. According to the literature data [10], there are three features of the HDDR process that allow for this assumption: (1) The disproportionation reaction begins at the grain boundaries and moves toward the center of each original grain. (2) The alloy components have a high diffusion rate due to the presence of hydrogen in the alloy. (3) The HDDR process homogenizes the alloy. According to this study, the disproportionation reaction occurs in the RE-rich phase and in a small volume of the main ferromagnetic phase, R_2_Fe_14_B. Because the reaction begins at the grain boundaries, the near-boundary regions of the R_2_Fe_14_B grains undergo disproportionation and recombination, becoming homogeneous. Even when HDDR transformations were not revealed in the R_2_Fe_14_B phase grains, the presence of hydrogen increased the diffusion rate of the alloy elements and increased the homogeneity of the R_2_Fe_14_B phase grains.

Application of the HDDR process for treatment of the R-Fe-B magnets under a hydrogen pressure of 20–30 kPa is acceptable for grinding their microstructure and obtaining anisotropic magnets. When these low pressures are applied during the disproportionation stage, the reaction time becomes a critical parameter.

Increasing the hydrogen pressure to 50 kPa during disproportionation results in a notable variation in the phase–structure state of sintered magnets after HDDR treatment. These variations depend on the treatment parameters and the depth of the samples. The main peculiarity is the complete disproportionation of the ferromagnetic phase near the sample surface. As a result, an isotropic ferromagnetic phase exists at depths of at least 70 µm from the sample surface after recombination. An anisotropic ferromagnetic phase was revealed at a depth of 500 µm. Thus, the DoT depends on depth and increases from the surface to the center of the sample, significantly influencing the sample’s overall texture. Except for one result obtained after HDDR, in which the HD stage was carried out at 785 °C for 4.5 h, and the DR stage was carried out at 850 °C, magnets treated at 50 kPa exhibited the lowest DoT (Figure 14). In this case, the DoT was slightly below the highest value obtained in this study. Additionally, applying a hydrogen pressure of 50 kPa during HDDR treatment revealed that the DoT depends on the recombination temperature. In particular, the DoT increases with an increasing DR temperature.

The newly obtained results provide a wide range of HD stage parameters, namely, hydrogen pressure from 20 to 50 kPa, temperatures from 700 to 785 °C, dwell time from 3 to 11 h, and DR stage temperature from 750 to 850 °C. Within this range, HDDR-treated sintered magnets are anisotropic. We can confidently conclude that there is significant room for optimizing the recombination stage to improve magnetic properties. This research is ongoing.

**Figure 16:**
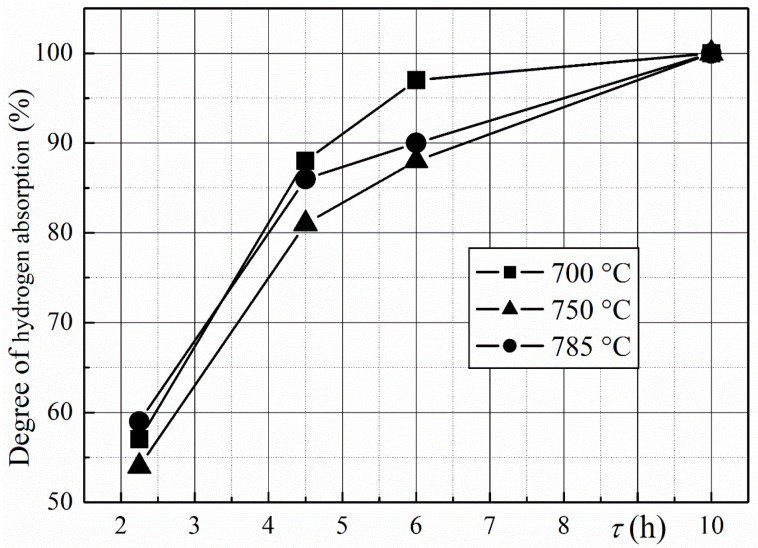
Relative amount of hydrogen absorbed by R-Fe-B magnets versus disproportionation reaction interaction time and temperature; hydrogen pressure of 50 kPa.

**Figure 17:**
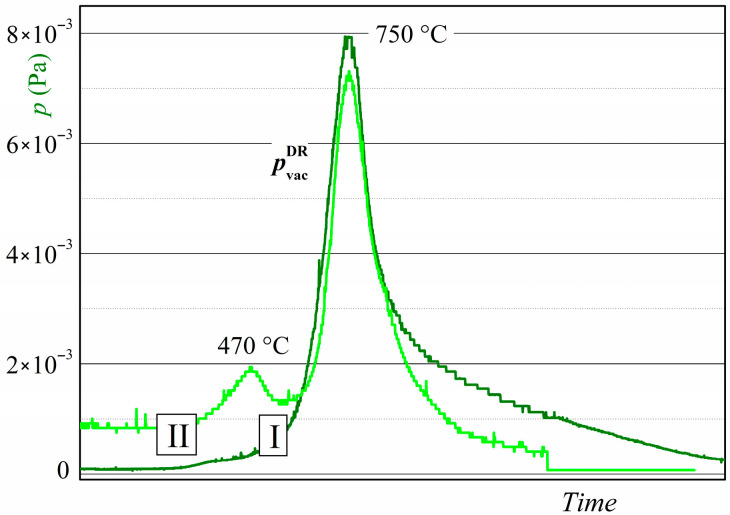
Hydrogen release curves in a vacuum during the DR stage ($p_{vac}^{DR}$). Curves I and II represent single- and double-peak curves, respectively.

The results presented here, namely those obtained at a hydrogen pressure of 50 kPa, reveal new details about the formation process of rare earth metal hydrides during the disproportionation stage of the HDDR process. This can be elucidated by comparing the XRD data with the amount of hydrogen absorbed by the samples during the disproportionation stage—estimated using measurements of hydrogen pressure changes (Figure 16)—and with the hydrogen release during the DR stage (Figure 17). According to the XRD data, the main ferromagnetic phase nearly completely decomposes into RH_2–3_ and α-Fe after the samples interact with hydrogen for 4.5 h (see, for example, Figure 9 or Figure 11). In this case, the material absorbs approximately 80–88% of the maximum possible amount of hydrogen (Figure 16). If the disproportionation reaction time increases to 6 h or more, the materials continue to absorb the hydrogen. We hypothesize that this absorption is caused by increasing hydrogen content in the rare earth hydride. To address this hypothesis, we consider two types of hydrogen desorption curves obtained here (Figure 17) and the literature data [51].

As evidenced by the literature, the rare earth metal hydrides (RH_~3_) exhibit two-peak decomposition behavior in both the single-phase state materials [52] and during the DR stage of the HDDR process when these hydrides are in a mixture of RH_2–3_, α-Fe, and Fe_2_B phases [51]. According to these data, the low-temperature peak corresponds to the partial decomposition of the rare earth hydrides, resulting in a change in composition from RH_~3_ to RH_~2_. The high-temperature peak is caused by the complete decomposition of the hydride, represented by the reaction RH_~2_ → R. In light of the two stages of the hydride decomposition and the exceedingly long disproportionation reaction dwell range, it is evident why the two types of hydrogen release curves were obtained during DR in this research (Figure 17). In the case of the single-peak curve, the release of hydrogen from the RH_~2_ hydride is observed. The double-peak curve exhibits the two stages of the decomposition of the RH_~3_ hydride. Therefore, it can be assumed that the main part of the ferromagnetic phase decomposes after the interaction of the magnet with hydrogen for a relatively short reaction time (e.g., for 4.5 h, hydrogen pressure of 50 kPa). In this case, the RH_~2_ hydride is formed, and a single-peak curve is observed during the DR stage. If the disproportionation reaction duration is longer, hydrogen absorption is associated with an increase in the hydrogen content of the rare earth hydride RH_~2+x_, where x ≤ 1. If this hydride forms, a double-peak curve is observed during the DR stage.

In light of the presented results, the application of the HDDR process in a wider range of low hydrogen pressures can be concluded to be a feasible approach for the post-sintering treatment of Nd-Fe-B-type magnets.

However, it is evident that the HDDR parameters need to be optimized, namely, the temperature and duration of the disproportionation reaction. Additionally, additional treatment is necessary to adjust the microstructure of the magnets and achieve high magnetic properties. Specifically, forming a non-ferromagnetic phase network around ferromagnetic phase grains is necessary to adjust the microstructure and achieve higher coercivity.

## 5. Conclusions

We demonstrated the potential of using the solid HDDR process as a post-sintering treatment for plate-shaped (Nd,Pr,Gd)-Fe-type magnets with a thickness of ~2 mm to obtain anisotropic magnets.

The treatment was performed at a hydrogen pressure within the range of 10–50 kPa with disproportionation and recombination temperatures of 700–785 °C and 750–850 °C, respectively. The treatment time ranged from 3 to ≤11 h during the HD stage. The solid HDDR treatment for grinding the microstructure of sintered magnets is acceptable within the hydrogen pressure range of 20–50 kPa due to the relatively short treatment time. Treatment at a lower hydrogen pressure of 10 kPa is preferable for achieving more textured microstructures in the treated magnets by homogenizing the near-boundary areas of the ferromagnetic phase grains.

The use of low-hydrogen-pressure and low-temperature parameter ranges for HDDR treatment provides an opportunity to regulate the disproportionation reaction rate with greater precision, which in turn affects the phase composition and degree of texture of materials. This study presents evidence for the texture memory mechanism, which is based on the idea that the presence of ferromagnetic phase remnants after the disproportionation reaction is a cause of texture formation in HDDR-treated materials. This connection has been confirmed multiple times for different hydrogen pressure values.

The results on the phase–structure state of sintered magnets after solid HDDR treatment indicate a wide range of process parameter options. In particular, temperature and reaction time can range from 700 to 785 °C and from 3 to 8 h, respectively, to produce anisotropic magnets with a fine microstructure.

## Data Availability

The original contributions presented in this study are included in this article; further inquiries can be directed to the corresponding author.
